# Fluorohydrocarbon Plasma Functionalization of Polyurethane Surfaces: Bacterial Adhesion and Cell Response

**DOI:** 10.3390/polym18091097

**Published:** 2026-04-30

**Authors:** Kamil Drożdż, Paulina Chytrosz-Wróbel, Divya Kumar, Karolina Zając, Andrzej Kotarba, Monika Brzychczy-Włocha

**Affiliations:** 1 Department of Molecular Medical Microbiology, Faculty of Medicine, Jagiellonian University Medical College, 31-121 Krakow, Poland; kamil.drozdz@doctoral.uj.edu.pl; 2 J. Heyrovský Institute of Physical Chemistry of the Czech Academy of Sciences, Dolejškova 2155/3, 182 23 Prague, Czech Republic; paulina.chytrosz@doctoral.uj.edu.pl; 3 Faculty of Chemistry, Jagiellonian University, 31-007 Krakow, Poland; divya.kumar@doctoral.uj.edu.pl (D.K.); karolina.zajac@doctoral.uj.edu.pl (K.Z.); kotarba@chemia.uj.edu.pl (A.K.)

**Keywords:** polyurethane, PU, low-temperature plasma, C_3_H_2_F_4_ plasma, bacterial adhesion, biocompatibility

## Abstract

Polyurethanes (PUs) are widely used in biomedical applications; however, their surface properties critically determine bacterial colonization and cell response. In this study, medical-grade PU films were modified using low-pressure C_3_H_2_F_4_ plasma (50 W, 300 s, 0.2 mbar), and the resulting changes in surface chemistry, wettability, topography, bacterial adhesion, and cell compatibility were evaluated. X-ray photoelectron spectroscopy (XPS) analysis confirmed the incorporation of fluorine-containing groups (CF_2_, CF_3_) and the appearance of an F 1s signal at ~688.3 eV. Plasma treatment increased the water contact angle from 92.6° ± 5.6° to 97.9° ± 3.1° and elevated the root mean square (RMS) surface roughness (Sq) from 39.0 nm to 77.3 nm. Surface free energy slightly decreased after plasma treatment due to reductions in both polar and dispersive components. Quantitative adhesion assays revealed strain-dependent effects. For *S. aureus* DSM 4910, *S. epidermidis* DSM 28319, and *P. aeruginosa* DSM 22644, no consistent reduction in adhesion was observed on plasma-treated surfaces. In contrast, *E. coli* DSM 18039 demonstrated significantly higher adhesion on modified PU at all incubation times, reaching 5.96 ± 0.44 logCFU/mL after 240 min compared to 5.05 ± 0.27 log colony-forming units per milliliter (logCFU/mL) on unmodified PU. Fluorescence microscopy confirmed increased surface coverage by *E. coli* on fluorinated samples. Biocompatibility studies using A549 cells showed no cytotoxic effects. Cell spreading area remained comparable between surfaces (1188.6 vs. 1185.1 µm^2^; *p* = 0.958). However, cells on plasma-treated PU exhibited reduced major axis length (38.6 vs. 46.7 µm; *p* < 0.001) and decreased focal adhesion area (8.88 vs. 10.94 µm^2^; *p* = 0.002), indicating moderate alterations in cell morphology without compromised viability. These results demonstrate that C_3_H_2_F_4_ plasma fluorination moderately increases PU hydrophobicity and nanoscale roughness, induces strain-dependent changes in bacterial adhesion—particularly enhancing *E. coli* colonization—while fully preserving mammalian cell viability and showing no cytotoxic effects of the modified surface.

## 1. Introduction

Polyurethanes (PUs) are segmented polymers containing urethane groups [–O–CO–NH–], formed by the reaction of diisocyanates with polyols. Due to the presence of both “hard” (isocyanate-based) and “soft” (polyol-based) segments, PUs combine high elasticity with excellent mechanical strength. They exhibit outstanding mechanical properties—such as high tensile and abrasion resistance, durability, and resilience—while maintaining good biocompatibility and hemocompatibility [1,2]. In medicine, PUs are widely used for the production of catheters, circulatory implants (e.g., artificial hearts and valves), prostheses, anti-adhesive coatings, as well as flexible dressings and intelligent bioindicator connectors [3,4]. The variable surface hydrophilicity of PU and the possibility of chemical modification (e.g., introducing hydroxyl or amino groups) enable fine-tuning of biomaterial–tissue interactions and improving implant acceptance by the host organism [2,5,6].

Despite their extensive clinical use, polyurethane-based indwelling devices remain susceptible to microbial colonization and biofilm-associated infections, which constitute a major complication of venous and urological catheters [7]. Medical device-associated infections represent a substantial fraction of hospital-acquired infections, and rapid bacterial adhesion followed by biofilm formation enables persistence of infection and increased tolerance to antibiotics and host defenses [8]. Importantly, biofilm-related catheter infections (e.g., catheter-associated urinary tract infections and central line-associated bloodstream infections) remain clinically prevalent and are linked to the early stages of surface colonization [9]. These considerations motivate the development of polyurethane surface-engineering strategies that can reduce early bacterial attachment while maintaining acceptable cytocompatibility [9].

One of the methods used to modify the surface of polyurethanes is low-temperature plasma treatment. Plasma is an ionized gas generated, for example, in radio-frequency discharges or at atmospheric pressure, in which electrons possess high energy while the bulk gas remains relatively cool. Such plasma contains electrons, ions, free radicals, and ultraviolet (UV) radiation, all of which interact with the material surface. The effects of plasma treatment, strongly depend on plasma parameters and source gas, include surface cleaning the attachment of new functional groups, etching and surface deposition. These processes can significantly alter surface properties—such as increasing hydrophilicity through oxidation or enhancing surface roughness—which in turn modifies wettability, surface energy, adhesion, and biocompatibility [10,11]. By adjusting the discharge parameters (gas type and composition, power, exposure time), plasma treatment provides a controlled and chemically clean (reagent-free) approach to modifying a thin surface layer of a biomaterial [12].

However, reported correlations between plasma parameters, the resulting surface chemistry, and biological performance are often scattered across the literature because different plasma sources and treatment conditions are used, making direct comparison difficult [13]. Moreover, improving wettability and cell response by oxygen plasma can simultaneously increase staphylococcal adhesion on polyurethane, highlighting the trade-off between cytocompatibility and resistance to microbial colonization [8].

Fluorine plasma (generated using fluorine-containing gases such as tetrafluoromethane CF_4_ or compounds like C_3_H_2_F_4_) leads to the incorporation of fluorine atoms into the outermost layer of the polyurethane surface. As a result, C–F, –CF_2_–, and –CF_3_ bonds are formed on the material surface. Because carbon–fluorine bonds are weakly polar and chemically inert, fluorinated surfaces become highly hydrophobic and exhibit reduced surface energy [14]. In practice, this translates into a significant increase in the water contact angle (the surface repels water) and a reduction in the adhesion of water and other fluids. Enhanced hydrophobicity limits the adsorption of proteins, bacteria, and cells on the PU coating, giving the material anti-adhesive properties. Fluorinated coatings are often used as “non-stick” surfaces—for example, for covering vascular stents or even ship hulls to reduce biofilm formation and fouling [14,15,16].

Therefore, there is a need for integrated studies that relate fluorocarbon-plasma-induced surface chemistry (including fluorocarbon (CFx) formation) to wettability and the early-stage response of both clinically relevant bacteria and mammalian cells under strictly comparable experimental conditions [13].

In this work, we investigate low-temperature C_3_H_2_F_4_ plasma treatment of medical-grade polyurethane and correlate X-ray photoelectron spectroscopy (XPS)-derived chemical states (C 1s, O 1s, F 1s) with wettability and biological response, aiming to clarify how fluorocarbon deposition influences bacterial attachment and cell–material interactions [14].

## 2. Materials and Methods

### 2.1. Preparation and Functionalization of the Material

Polyurethane films with a thickness of 100 μm, produced from medical-grade amorphous aromatic polyether-based polyurethane, were obtained from American Polyfilm, Inc. (Branford, CT, USA). Before surface treatment, the films were cut into discs with a diameter of 14 mm, cleaned with 2-propanol (Avantor, Gliwice, Poland), and subsequently air-dried. After drying, the polyurethane was treated with low-pressure C_3_H_2_F_4_ plasma using a Diener Electronic plasma surface modification system. The process was carried out under the following conditions: plasma power set to 50, exposure time: 300 s and a C_3_H_2_F_4_ partial pressure maintained at 0.2 mbar.

### 2.2. X-Ray Photoelectron Spectroscopy (XPS)

The surface chemical composition was examined by X-ray Photoelectron Spectroscopy (XPS) using an ultra-high-vacuum setup equipped with a SES R4000 analyzer (Gammadata Scienta, Uppsala, Sweden). Measurements were conducted with a monochromatic Al Kα radiation source (1486.6 eV) operating at 350 W. The spectra were collected at a take-off angle of 90°, while the pressure in the analysis chamber was maintained below 5 × 10^−9^ mbar. Survey spectra were acquired with a pass energy of 100 eV. High-resolution spectra were recorded at a pass energy of 20 eV to improve spectral resolution. Charge compensation was applied using a low-energy electron flood gun. XPS measurements were performed on three independently prepared polyurethane samples for each surface variant. The presented spectra are representative, and no significant differences were observed between independently prepared samples. Data were processed using CasaXPS software (version 2.3.15, Casa Software Ltd., Teignmouth, UK). A Shirley-type background subtraction was applied prior to spectral deconvolution. The binding energy scale was calibrated using the C 1s signal corresponding to C–C/C–H bonds at 285.0 eV as an internal reference [7]. The atomic surface composition of the polyurethane was calculated from the integrated peak areas of the C 1s and O 1s core levels using appropriate sensitivity factors provided in the CasaXPS database.

### 2.3. Contact Angle and Surface Free Energy Calculations

The wettability of both untreated and fluorine plasma–modified surfaces was assessed using a Surftens Universal device (OEG GmbH, Frankfurt, Germany) positioned inside a temperature-controlled chamber. Static contact angles of water were determined with the Surftens 4.3 software (OEG GmbH, Frankfurt, Germany) operating under the Windows environment. Surface free energy (SFE) was calculated using the Owens–Wendt approach based on contact angle measurements with distilled water and diiodomethane (CH_2_I_2_) as polar and dispersive probe liquids, respectively [17,18]. For each sample, five independent measurements were performed for each probe liquid droplets of 2.5 μL volume.

### 2.4. Atomic Force Microscopy (AFM)

Surface topography of both pristine and plasma-treated samples was analyzed using atomic force microscopy (AFM) operated in contact mode (Qi mode). Measurements were performed with a JPK NanoWizard 4 XP system (Bruker Nano GmbH, Berlin, Germany) coupled to an Olympus optical microscope and mounted on an Accurion i4 (Accurion GmbH, Göttingen, Germany) active vibration isolation platform. All experiments were carried out in air under ambient conditions inside an acoustic enclosure, employing a Bruker TESPG-V2 silicon probe doped with antimony. The acquired AFM data were processed and evaluated using JPK Data Processing 7.0.162 software. The root mean square (RMS) surface roughness (Sq) was determined from AFM height data using Gwyddion 2.69 (Czech Metrology Institute, Brno, Czech Republic), by analyzing five randomly selected 5 × 5 µm^2^ regions within each 20 × 20 µm^2^ scan.

### 2.5. Adhesion of Bacteria

Bacterial strains and culture preparation: The study utilized two Gram-positive bacterial strains—*Staphylococcus aureus* DSM 4910 (Deutsche Sammlung von Mikroorganismen und Zellkulturen (DSMZ), Braunschweig, Germany) and *Staphylococcus epidermidis* DSM 28319 as well as two Gram-negative strains—*Escherichia coli* DSM 18039 and *Pseudomonas aeruginosa* DSM 22644. The frozen cultures were reactivated by streaking onto agar plates containing 5% sheep blood (Becton Dickinson, Franklin Lakes, NJ, USA) and incubating at 37 °C for 24 h. For experimental purposes, a single colony from each strain was inoculated into 100 mL of tryptic soy broth (TSB; Becton Dickinson) and cultured for 18 h at 37 °C.

Adhesion assay procedure: The adhesion experiments were carried out in phosphate-buffered saline (PBS; Chempur^®^, Piekary Śląskie, Poland), providing conditions under which the natural surface charge of bacterial cells is preserved. Following incubation, 18 h cultures were washed three times with PBS by centrifugation at 3000 rpm for 4 min to expose the native bacterial surface charge. Static adhesion tests were performed in 24-well plates (Nest Biotechnology Co., Wuxi, China). The samples were sterilized under UV light for 20 min and subsequently secured at the bottom of the wells using sterile quartz rings. Prior to each microbiological experiment, sterility of the polyurethane samples was verified by incubation in sterile TSB medium for 24 h to confirm the absence of microbial contamination.

The biomaterial discs were incubated in 0.5 mL of a 10^6^ colony forming units per milliliter (CFU/mL) bacterial suspension for 30, 60, and 240 min, followed by triple PBS rinsing to remove loosely attached microorganisms.

Quantification and microscopic analysis of bacterial adhesion: The number of bacteria on the surface of biomaterials was assessed using the serial dilution method. For this purpose, 0.25% trypsin (BD DifcoTM) diluted in PBS was used and bacteria were mechanically washed from the surface of the polyurethanes. Then, serial 10-fold dilutions of the resulting bacterial suspension were made and 100 μL were seeded on tryptic soybean agar (TSA) medium (Dickinson) for each dilution in duplicate. Plates were incubated for 24 h at 37 °C. After this time, the bacterial colonies were counted, and the results are given in colony forming unit (CFU) per mL. The number of adherent bacteria on the biomaterial surfaces was quantified using the serial dilution technique. To detach adherent bacteria, 1 mL of sterile PBS was applied, and the sample surface was rinsed by repeated aspiration and dispensing of the liquid using a pipette (approximately 25 times over the entire sample surface) to ensure efficient recovery of attached bacterial cells. The obtained bacterial suspensions were then subjected to a series of tenfold dilutions, and 100 μL of each dilution was plated in duplicate onto TSA. The plates were incubated for 24 h at 37 °C. After incubation, colony numbers were determined, and the results were expressed as colony-forming units (CFU) per mL.

The area occupied by bacteria on the polyurethane surface was also evaluated. For this purpose, an Olympus BX63 fluorescence microscope (Olympus Corporation, Tokyo, Japan) with UPlanXApo20x/0.80 objective was used. The bacteria were stained with propidium iodide (PI) according to the manufacturer’s recommendations (Life Technologies, Waltham, MA, USA). The area occupied by bacteria (%) was assessed using the ImageJ 1.54i software (National Institutes of Health, Bethesda, MD, USA) based on fluorescence images obtained from the tetramethylrhodamine (TRITC) channel (red channel–stained bacteria). All experiments were conducted in triplicate.

### 2.6. Biocompatibility

Cell line cultures: To evaluate biocompatibility and viability, the A549 cell line derived from human lung epithelium (CRL-185™, American Type Culture Collection) was used. The human A549 cell line (alveolar basal epithelial cells) was selected for cytocompatibility testing because it represents an epithelial phenotype and is widely used to assess cell attachment, morphology, and viability on biomaterial surfaces in vitro studies.

The cells were cultured in Dulbecco’s Modified Eagle Medium (DMEM) Gibco (Thermo Fisher Scientific, Waltham, MA, USA) enriched with 10% fetal bovine serum (Gibco) and kept at 37 °C in a humidified 5% CO_2_ atmosphere. To prevent microbial contamination, the culture medium was supplemented with ZellShield^®^ (Minerva Biolabs GmbH, Burlington, VT, USA), an antibiotic complex composed of natamycin, ciprofloxacin, and clindamycin.

Live/dead cell line staining with FDA and PI: Cell viability was evaluated using Fluorescein Diacetate (FDA; Sigma-Aldrich, St. Louis, MO, USA) and Propidium Iodide (PI; Sigma). For the live/dead assay, A549 cells were seeded in 24-well plates (Nest) at a density of 3 × 10^4^ cells per well in 1 mL of complete culture medium. The biomaterial samples were then brought into contact with the cells and incubated for 24 h. To prepare the staining solutions, FDA (5 mg/mL) was diluted 1:1250 and PI (2 mg/mL) 1:40 in PBS. After incubation, the samples were gently rinsed with PBS, mounted with a coverslip in PBS, and the stained cells were subsequently examined under an Olympus BX63 fluorescence microscope using fluorescein isothiocyanate (FITC) (green, viable cells) and TRITC (red, dead cells) filter channels. All experiments were performed in triplicate to ensure reproducibility. Before testing, polyurethane samples were sterilized by UV irradiation for 20 min and subsequently held in place at the bottom of the wells using sterile quartz rings to maintain their position during incubation.

Cytoskeleton staining: Phalloidin and focal adhesion kinase (FAK) staining were used to evaluate the biocompatibility of the tested surfaces. The A549 cell line (CRL-185™, ATCC) was seeded into 24-well plates (Nest) at a density of 3 × 10^4^ cells per well in 1 mL of culture medium. Polyurethane samples were sterilized under UV light for 20 min and subsequently held in position using sterile quartz rings. The biomaterial surfaces were then incubated with the cells for 24 h. The Actin Cytoskeleton and Focal Adhesion Staining Kit (Merck Millipore, Burlington, VT, USA) was employed to visualize the actin filaments and FAK distribution. Cells were fixed with 4% paraformaldehyde (Sigma) in PBS for 15 min, rinsed twice with PBS containing 0.05% Tween-20 (Sigma), and then permeabilized with 0.1% Triton X-100 (Sigma) in PBS for 5 min. The procedure was followed by two further washes with PBS containing 0.05% Tween-20. Primary anti-vinculin antibodies diluted 1:500 in blocking solution were applied and incubated for 1 h at room temperature. Afterward, the cells were washed three times with wash buffer (10 min per wash). Double staining was conducted using TRITC-conjugated Phalloidin (1:500) and FITC-conjugated goat anti-mouse (Gt × Ms) antibodies (Millipore, Cat. No. AP124F) diluted 1:200 in PBS. Samples were incubated for 30 min, followed by three additional washes of 10 min each. In the final step, the nuclei were stained with 4′,6-diamidino-2-phenylindole (DAPI) for 5 min at room temperature and subsequently rinsed three times with wash buffer. The samples were mounted on microscope slides, covered with coverslips, and sealed with a drop of PBS. Fluorescence images were acquired using an Olympus BX63 microscope with DAPI, FITC, and TRITC channels corresponding to blue (DNA), green (FAK-100), and red (F-actin) fluorescence, respectively. Image analysis was performed in ImageJ software (version 1.53) to quantify the cell area, major axis length, and circularity. Circularity was calculated using the formula:

(1) $$Circularity=\frac{4\pi \cdot area}{{perimeter}^{2}}$$

where a value of 1.0 indicates a perfect circle. All experiments were conducted in triplicate.

The metabolic activity of A549 cells cultured on unmodified and C_3_H_2_F_4_ plasma–modified polyurethane surfaces was evaluated using the AlamarBlue reduction assay (Sigma–Aldrich, Merck KGaA, St. Louis, MO, USA).

Prior to cell seeding, polyurethane samples were sterilized by UV irradiation for 20 min and placed at the bottom of 24-well culture plates using sterile quartz rings to maintain their position during incubation. A549 cells were seeded at a density of 3 × 10^4^ cells per well in 1 mL of complete DMEM medium supplemented with 10% fetal bovine serum (Gibco) and ZellShield^®^ (Minerva Biolabs). Cells were cultured under standard conditions (37 °C, humidified atmosphere, 5% CO_2_) for 24 h in direct contact with the tested surfaces.

After incubation, the culture medium was replaced with fresh medium containing 10% AlamarBlue reagent and samples were incubated for 4 h at 37 °C to enable metabolic conversion of resazurin. Subsequently, 100 µL aliquots of the supernatant from each well were transferred to black 96-well plates and fluorescence intensity was measured using a microplate reader (SpectraMax Mini Multi-Mode Microplate Reader, Molecular Devices, San Jose, CA, USA). Cellular metabolic activity was expressed as the percentage of resazurin reduction calculated according to the following equation:

(2) $$\%\;resazurin\;reduction=\frac{S_{x}-S_{0}}{S_{100}-S_{0}}$$

where *S_x_* is the fluorescence intensity of the tested sample, *S_0_* represents the non-reduced AlamarBlue control (0% reduction), and *S_100_* corresponds to the fully reduced AlamarBlue control obtained after autoclaving (100% reduction reference).

### 2.7. Statistical Analysis

Statistical analysis was conducted using the IBM SPSS Statistics 29.0.2.0 (20) package. The normality of the distribution of continuous variables was checked using the Shapiro–Wilk test. Since the data did not meet the assumptions of normality, comparisons between unmodified and plasma-treated samples were performed using the non-parametric Mann–Whitney U test. For variables showing normal distribution, comparisons between groups were performed using Student’s *t*-test. When the assumption of homogeneity of variances was not met, Welch’s correction was applied. Results were considered statistically significant when *p* < 0.05.

## 3. Results and Discussions

### 3.1. XPS

**Figure 1:**
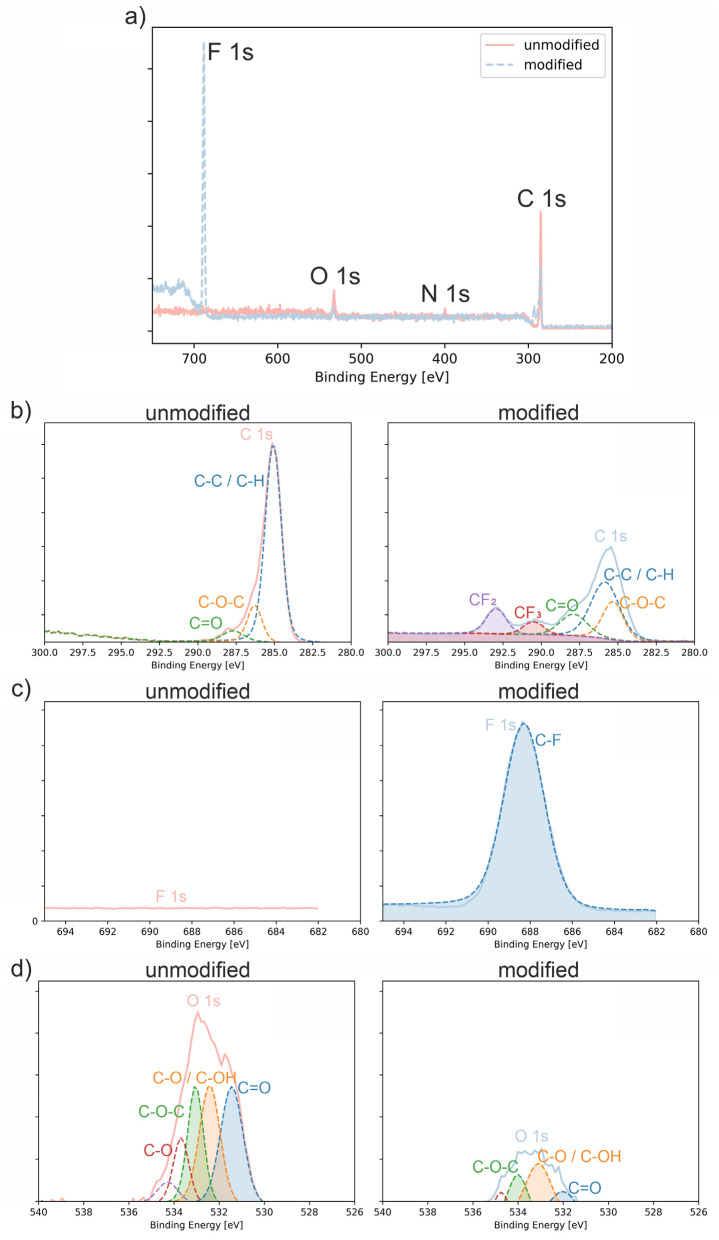
XPS survey spectrum (**a**) and high-resolution narrow scans for polyurethane surfaces: (**b**) C 1s region, (**c**) F 1s region, and (**d**) O 1s region, presented for both: unmodified and plasma-treated samples (modified).

To examine plasma-induced changes in surface chemistry, polyurethane samples were analyzed by X-ray Photoelectron Spectroscopy (XPS) after C_3_H_2_F_4_ plasma treatment (Figure 1). In the survey spectra of the unmodified polyurethane, three characteristic signals were observed: C 1s (~285.2 eV), O 1s (~532.4 eV), and N 1s (~399.6 eV). Following C_3_H_2_F_4_ plasma treatment, these peaks shifted slightly (C 1s ~286.0 eV, O 1s ~533.2 eV, N 1s ~400.8 eV), and an additional F 1s signal appeared at ~688.4 eV, indicating the presence of fluorine-containing species on the surface [19].

The high-resolution C 1s spectra of the unmodified polyurethane exhibited three characteristic components attributed to C–C/C–H (285.1 eV), C–O–C (286.3 eV), and C=O (287.7 eV) (Table 1). After C_3_H_2_F_4_ plasma treatment, these peaks exhibited systematic chemical shifts, indicating changes in their local electronic environment. In addition, two new components were observed at ~290.5 eV and ~292.9 eV, which were assigned to CF_2_ and CF_3_ groups, respectively [20,21]. The assignment of the high-binding-energy components to CF_2_ and CF_3_ groups is consistent with previously reported XPS analyses of plasma-deposited fluorocarbon films, where the C1s envelope is typically resolved into CF_3_, CF_2_, CF, and C–C contributions [22]. A distinct F 1s signal was detected at ~688.3 eV. The C–F contribution within the C 1s region partially overlapped with neighboring oxygen-containing carbon components, as reported for fluorinated polymers [23]. The appearance of CF2 and CF3 components confirms the formation of a fluorocarbon-enriched surface layer on the polyurethane surface, which is further supported by the significant decrease in the oxygen atomic concentration after plasma treatment (from 11 at.% to 3 at.%) (Table 2).

A closer inspection of the O 1s region revealed several oxygen-containing components that were present both before and after plasma treatment. For the unmodified polyurethane, the O 1s envelope consisted primarily of signals attributed to carbonyl groups (C=O, ~531.4 eV), C–O/C–OH (~532.4 eV), and ether-type C–O–C structures (~533.0 eV). Additional minor contributions were observed at higher binding energies (533.7–534.3 eV), which are typically associated with more oxidized oxygen environments. Following C_3_H_2_F_4_ plasma treatment, all O 1s components exhibited a systematic chemical shift of approximately +0.6–0.8 eV, yielding peaks at ~532.0 eV (C=O), ~533.1 eV (C–O/C–OH), and ~534.0 eV (C–O–C), along with two higher-energy contributions at ~534.8 eV and ~535.5 eV [21,24]. Considering the simultaneous appearance of CF_2_ and CF_3_ components in the C 1s spectrum, these high-energy features are attributed to oxygen atoms in a strongly electron-deficient environment induced by neighboring CF_x_ groups, rather than formation of a dominant new oxygen chemical state.

After plasma treatment with C_3_H_2_F_4_, new high-binding-energy components corresponding to CF_2_ and CF_3_ groups appear in the C 1s spectrum, confirming deposition of fluorocarbon species. Concurrently, the oxygen atomic concentration decreases markedly, indicating enrichment of the surface with fluorocarbon species. In the O 1s region, the main component remains centered around ~533 eV (C–O), while a minor high-binding-energy contribution (~535.5 eV) is observed in the modified sample. This shift toward higher binding energies is attributed to the strong electron-withdrawing effect of neighboring CF_x_ groups.

C_3_H_2_F_4_ plasma treatment resulted in clear modifications of the polyurethane surface chemistry, as evidenced by the appearance of CF_2_ and CF_3_ components in the C 1s region and the detection of the F 1s signal. The observed binding energies are consistent with previously reported values for fluorinated polymer surfaces [21,23,24].

### 3.2. Contact Angle and Surface Free Energy Calculations

**Figure 2:**
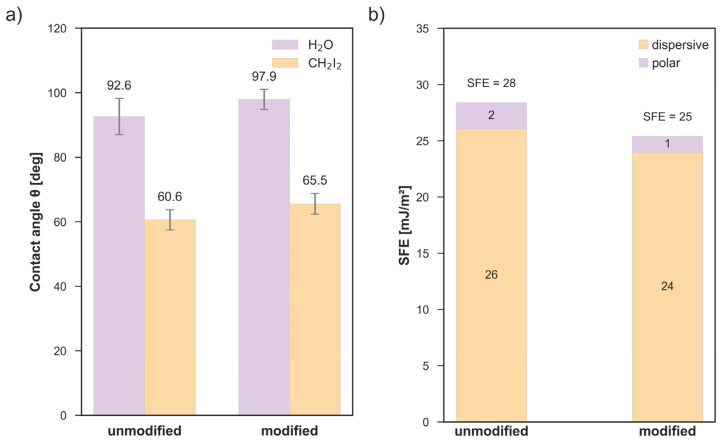
(**a**) Mean contact angle values (θ) with standard deviation measured using distilled water (H_2_O) and diiodomethane (CH_2_I_2_) for untreated (unmodified) and C_3_H_2_F_4_ plasma-treated (modified) polyurethane surfaces. (**b**) Surface free energy (SFE) and its dispersive and polar components calculated using the Owens–Wendt method for unmodified and plasma-treated polyurethane samples.

Surface modification influenced the wettability of polyurethane, as assessed by static water contact angle measurements. The unmodified material exhibited hydrophobic behavior. After C_3_H_2_F_4_ plasma treatment, the contact angle increased on average by approximately 5°, indicating low surface hydrophobization (Figure 2a). A similar trend was observed for diiodomethane (CH_2_I_2_), for which the contact angle increased from 60.6° ± 3.1° for the unmodified surface to 65.5° ± 3.2° after plasma treatment, confirming a slight decrease in dispersive interactions at the material surface (Figure 2a). The observed difference remained within the range of experimental variability.

Consistent with these observations, the surface free energy (SFE) calculated using the Owens–Wendt approach slightly decreased from 28 mJ/m^2^ for the unmodified polyurethane to 25 mJ/m^2^ after modification. This change resulted from small reductions in both the dispersive component (from 26 to 24 mJ/m^2^) and the polar component (from 2 to 1 mJ/m^2^), indicating that the applied plasma treatment induced only minor changes in the energetic structure of the polyurethane surface (Figure 2b).

According to literature data, unmodified polyurethane typically exhibits water contact angles close to 90° [2,25], which is consistent with the value observed in the present study (92.6° ± 5.6°).

Fluorine-based plasma treatments are widely applied to reduce surface free energy and increase hydrophobicity of polymeric materials [26]. This observation is consistent with the AFM results obtained in the present study, which indicate preservation of the global surface morphology accompanied by nanoscale roughness changes.

In some cases, more pronounced fluorination results in contact angles exceeding 100°, as reported for fluorinated polyurethane (109.0° ± 2.7°) [27] or highly hydrophobic fluoropolymer films such as PFA and PTFE [28]. In contrast, the increase observed in the present study was moderate, indicating that the applied plasma conditions induced only partial surface hydrophobization.

This effect is particularly relevant for fluorocarbon plasma-modified polyurethane surfaces, where modest reductions in both polar and dispersive components of surface free energy primarily reflect the incorporation of CFx functional groups rather than major changes in surface polarity. As a consequence, such modifications typically lead to strain-specific rather than universal reductions in bacterial adhesion, especially when comparing Gram-positive species (*S. aureus*, *S. epidermidis*) and Gram-negative species (*E. coli*, *P. aeruginosa*), which differ substantially in outer surface composition and interaction mechanisms with hydrophobic polymer substrates [29].

### 3.3. Atomic Force Microscopy

**Figure 3:**
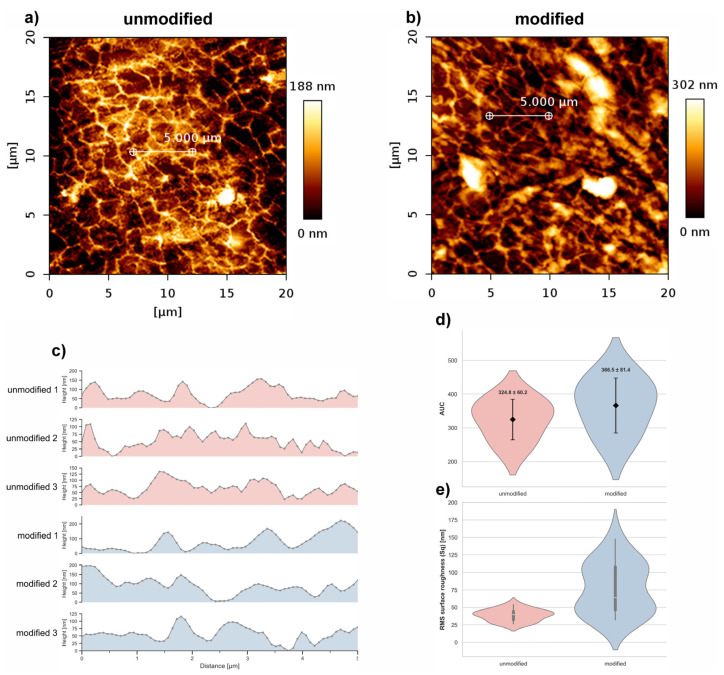
Comparison of surface morphology between unmodified (**a**) and C_3_H_2_F_4_ plasma–treated (modified) (**b**) polyurethane visualized by AFM. Panel (**c**) presents representative surface height profiles measured along 5 μm lines extracted from three independent AFM scan areas for each surface type, panel (**d**) presents the mean area under the curve (AUC) values with standard deviation (mean ± SD) calculated from the corresponding AFM profiles. Panel (**e**) shows a box-plot of the RMS surface roughness (Sq) values calculated from five randomly selected 5 × 5 µm^2^ regions (boxplot).

The effect of C_3_H_2_F_4_ plasma modification on the surface topography of polyurethane was evaluated based on AFM images collected from a 20 µm × 20 µm scan area for both unmodified and plasma-treated samples (Figure 3a,b).

AFM analysis showed that the overall topography of the polyurethane surface remained similar after C_3_H_2_F_4_ plasma treatment. Height profiles and roughness distributions showed only minor differences between unmodified and modified samples, with no clear tendency toward either smoothing or roughening. Additionally, to quantitatively assess the changes, the area under the curve (AUC) of the surface profiles was calculated for three representative cross-sections (Figure 3c). The mean AUC values were 324.8 ± 60.2 for the unmodified surface and 366.5 ± 81.4 for the plasma-treated one. The RMS roughness parameter (Sq) increased from 39.0 nm (Q1–Q3: 32.2–44.6 nm) for the unmodified sample to 77.3 nm (Q1–Q3: 46.4–108.1 nm) after C_3_H_2_F_4_ plasma treatment.

Plasma fluorination proceeds differently from oxidative or aminating treatments. The formation of C–F bonds leads to surface passivation, reduced chemical reactivity, and increased hydrophobicity [30,31,32]. In the present study, AFM analysis showed that the global surface profile remained comparable between treated and untreated samples, while the RMS roughness increased from 39.0 nm to 77.3 nm. This suggests that C_3_H_2_F_4_ plasma induced nanoscale surface irregularities without extensive macroscopic erosion.

Overall, C_3_H_2_F_4_ plasma can be considered a mild modification method that preserved the global morphology of polyurethane while inducing nanoscale rearrangements and a moderate increase in surface roughness, without altering the macroscopic surface profile.

### 3.4. Bacterial Adhesion Analysis: Serial Dilution and Plate Counts and Fluorescence Microscopy

**Figure 4:**
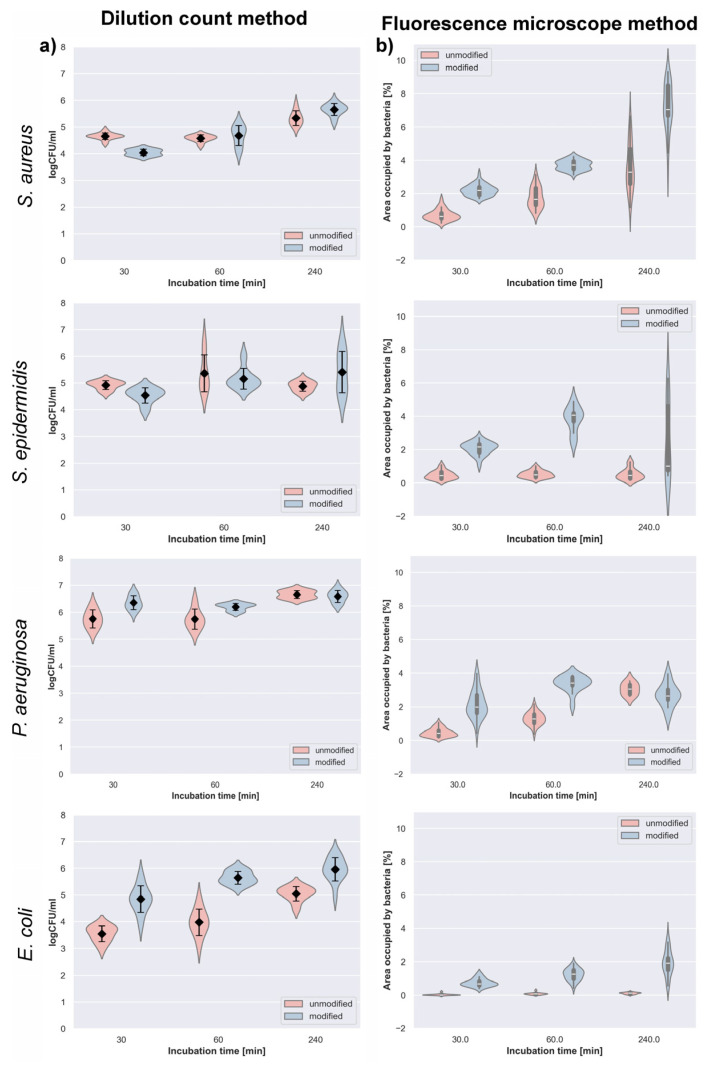
Quantitative assessment of bacterial adhesion on unmodified and plasma-treated polyurethane surfaces obtained by the serial dilution method (**a**) and fluorescence microscopy with propidium iodide (PI) staining (**b**). Both panels present data as violin plots; the mean (♦) and standard deviation are marked in (**a**), whereas (**b**) displays box plots illustrating data distribution with the median represented by the white line.

Serial dilution and plate counts: To evaluate the influence of plasma modification on bacterial attachment, quantitative analysis of viable cell counts (CFU) was performed for both unmodified and plasma-treated polyurethane surfaces. Four reference strains representing Gram-positive and Gram-negative bacteria were examined after 30, 60, and 240 min of incubation (Figure 4).

For *S. aureus*, the number of viable cells (logCFU) increased progressively with incubation time on both types of polyurethane. After 30 min, bacterial adhesion was lower on the plasma-treated surface (4.04 ± 0.12) than on the untreated one (4.65 ± 0.12). At 60 min, no notable difference between the surfaces was observed (4.67 ± 0.38 vs. 4.57 ± 0.13). However, after 240 min, the modified polyurethane exhibited a higher bacterial count (5.65 ± 0.23) compared to the unmodified material (5.33 ± 0.27). In the case of *S. epidermidis*, the number of viable bacteria (logCFU) generally increased with incubation time regardless of the surface type. After 30 min, slightly fewer cells adhered to the modified polyurethane (4.53 ± 0.29) compared with the unmodified surface (4.92 ± 0.17). After 60 min, the bacterial loads were nearly equivalent (5.15 ± 0.39 for modified and 5.36 ± 0.69 for unmodified polyurethane). Following 240 min of incubation, a higher mean bacterial count was found on the modified surface (5.40 ± 0.78) than on the unmodified one (4.88 ± 0.19). For *P. aeruginosa*, both unmodified and modified polyurethane surfaces supported high bacterial loads (logCFU) throughout the experiment. After 30 min, greater adhesion was detected on the plasma-modified material (6.35 ± 0.26) compared to the unmodified one (5.75 ± 0.34). At 60 min, the same tendency persisted (6.20 ± 0.13 vs. 5.74 ± 0.37). After 240 min, the bacterial counts on both surfaces were comparable—6.58 ± 0.22 for the modified and 6.66 ± 0.14 for the unmodified samples. Regarding *E. coli*, bacterial adhesion (logCFU) was consistently higher on the plasma-modified polyurethane surface across all incubation periods. After 30 min, the modified material showed a value of 4.85 ± 0.50, compared to 3.55 ± 0.30 for the unmodified one. After 60 min, adhesion increased further (5.65 ± 0.24 vs. 3.98 ± 0.49). At 240 min, the difference remained significant, with bacterial loads of 5.96 ± 0.44 for the modified and 5.05 ± 0.27 for the unmodified polyurethane.

Fluorescence microscope method: To visualize and quantify bacterial colonization, fluorescence microscopy with PI staining was applied. This method allowed assessment of surface coverage by live and dead bacterial cells on unmodified and plasma-modified polyurethane after different incubation times. The progression of *S. aureus* colonization was evident on both polyurethane types, with a pronounced increase in surface coverage over time. After 30 min, the modified polyurethane exhibited a median coverage of 2.20% (Q1–Q3: 1.90–2.38%), whereas the unmodified sample showed only 0.64% (Q1–Q3: 0.50–0.82%). By 60 min, adhesion intensified, reaching 3.72% (Q1–Q3: 3.46–3.94%) on the modified and 1.65% (Q1–Q3: 1.32–2.32%) on the unmodified surface. After 240 min, *S. aureus* formed a substantially denser layer on the plasma-modified polyurethane, covering 7.05% (Q1–Q3: 6.68–8.49%) of the area compared with 3.28% (Q1–Q3: 2.59–4.67%) for the untreated sample. In the case of *S. epidermidis*, a gradual increase in the percentage of surface occupation was observed for both materials, though more pronounced on the plasma-modified polyurethane. After 30 min, the modified surface reached a median coverage of 2.16% (Q1–Q3: 1.82–2.32%), compared to 0.44% (Q1–Q3: 0.25–0.64%) on the unmodified one. At 60 min, adhesion became more evident on the modified polyurethane (4.07%, Q1–Q3: 3.70–4.19%), whereas the unmodified surface showed minimal colonization (0.49%, Q1–Q3: 0.35–0.65%). After 240 min, adhesion on the modified surface displayed high variability (median 1.01%, Q1–Q3: 0.76–4.62%), while the unmodified polyurethane remained only slightly colonized (0.45%, Q1–Q3: 0.26–0.67%). For *P. aeruginosa*, the modified polyurethane exhibited enhanced early adhesion compared with the unmodified material. After 30 min, median surface coverage reached 2.00% (Q1–Q3: 1.64–2.70%) for the modified samples and 0.42% (Q1–Q3: 0.25–0.59%) for the unmodified ones. At 60 min, adhesion further increased on the modified surface (3.42%, Q1–Q3: 3.26–3.76%) while remaining lower on the untreated polyurethane (1.28%, Q1–Q3: 1.04–1.56%). After 240 min, both surfaces exhibited comparable coverage levels 2.64% (Q1–Q3: 2.42–3.03%) for the modified and 3.06% (Q1–Q3: 2.74–3.32%) for the unmodified material. In contrast, *E. coli* displayed persistently greater surface coverage on the plasma-modified polyurethane at all time points. After 30 min, the median coverage on the modified surface was 0.69% (Q1–Q3: 0.59–0.84%), while the unmodified polyurethane showed minimal colonization (0.02%, Q1–Q3: 0.01–0.04%). At 60 min, bacterial adhesion increased notably to 1.27% (Q1–Q3: 0.99–1.51%) on the modified material and 0.08% (Q1–Q3: 0.04–0.10%) on the unmodified one. Following 240 min of incubation, the modified samples exhibited a median coverage of 1.91% (Q1–Q3: 1.53–2.21%), compared with only 0.13% (Q1–Q3: 0.07–0.16%) for the unmodified polyurethane.

Bacterial adhesion to polymeric biomaterials is governed by a complex interplay between surface chemistry, surface energy, topography, and the physicochemical properties of the bacterial cell wall. Gram-negative rods such as *E. coli* and *P. aeruginosa* possess an outer membrane enriched in lipopolysaccharides (LPS). The presence of LPS renders their cell surface relatively hydrophilic and negatively charged, which limits their interactions with nonpolar, hydrophobic material [33,34]. Accordingly, studies on highly fluorinated and often micro/nanostructured surfaces have shown a pronounced decrease in Gram-negative bacterial adhesion after plasma treatment. It has been observed that the density of attached *P. aeruginosa* cells decreased with increasing water contact angle (up to 145°), reflecting enhanced surface hydrophobicity [35]. In the case of polypropylene (PP) modified by reactive ion etching (RIE) in a fluorine atmosphere, the resulting micro/nanofibrous structure exhibited extreme hydrophobicity (contact angle 155°), leading to a 99.6% reduction in *E. coli* adhesion [36]. Gram-positive cocci, such as *S. aureus* and *S. epidermidis*, have a different cell wall structure, lacking an outer membrane and containing a thick peptidoglycan layer with teichoic acids. The surface of many Staphylococcus species is more hydrophobic than that of Gram-negative bacteria, which promotes stronger adhesion to nonpolar materials. Some studies have shown that *S. aureus* preferentially adheres to hydrophobic surfaces, exhibiting stronger attachment to nonpolar than to polar materials [35]. However, more recent reports indicate that appropriate fluorine modification can also reduce the adhesion of Gram-positive bacteria, especially when combined with controlled surface topography. Xu and Siedlecki demonstrated that the biofilm-forming strain *S. epidermidis* RP62A colonized hydrophobic, textured polyurethane surfaces much less effectively than smooth ones [37]. Interestingly, when the same surfaces were rendered hydrophilic (by brief air plasma treatment), the effect was reversed—*S. epidermidis* adhesion increased on microtextured surfaces, while only submicron-scale textures partially retained anti-adhesive properties [37]. Overall, most studies confirm that properly designed fluorinated surfaces, especially those with micro/nanotextures reducing the contact area, also hinder the colonization of Gram-positive pathogens [37]. Similar effects were observed for fluorinated diamond-like carbon (F-DLC) coatings deposited by RF plasma onto UHMWPE substrates—in this case, adhesion of *S. aureus* and *S. epidermidis* was significantly lower compared to non-fluorinated coatings, which was attributed to the increased surface hydrophobicity [38]. Studies have also shown that modification with CF_4_/SF_6_ plasma does not increase surface toxicity toward bacteria but primarily reduces their attachment. In biofilm coverage assays, bacterial surface colonization was reduced by 50–90%, depending on the species and conditions, in favor of fluorinated surfaces compared to unmodified ones [35,37]. Fluorinated polyurethane surfaces are generally characterized by reduced surface free energy, which from the standpoint of adhesion thermodynamics means a reduced tendency to wetting and to form strong interactions with polar molecules, including water and biomolecules. Therefore, a bacterium settling on such a hydrophobic substrate encounters less favorable conditions for stable attachment. As reported in the literature, ultrahydrophobic surfaces exhibit anti-adhesive behavior mainly due to their low surface energy, which limits protein and cell adsorption [39]. In the present study, however, the contact angle increase remained moderate, which may explain why such a strong anti-adhesive effect was not observed for all tested strains. Therefore, the present results should not be directly compared with highly or superhydrophobic fluorinated surfaces reported in the literature, where substantially higher contact angles and hierarchical micro/nanostructures are typically responsible for anti-adhesive behavior.

In this range of surface energy changes, the effect on early bacterial adhesion is expected to be subtle and may easily be masked by experimental variability and the simultaneous contribution of other surface-related factors, such as surface chemistry and nanotopography; therefore, the interpretation of these differences should be made cautiously [13]. Importantly, for many polymeric systems the highest levels of bacterial adhesion are observed at intermediate hydrophobicity, typically corresponding to water contact angles around 90–95°, whereas only strongly hydrophilic or superhydrophobic surfaces tend to significantly reduce bacteria–surface interactions by limiting effective contact area or increasing repulsive interfacial forces [40].

This explains why the slight shift in water contact angle observed in the present study (from ~93° to ~98°) did not result in a consistent reduction in bacterial attachment and may even promote adhesion of selected strains. Moreover, the relationship between bacterial adhesion and surface hydrophobicity or surface free energy is frequently non-monotonic and strain-dependent, since attachment is strongly influenced by bacterial cell wall properties such as surface hydrophobicity, electrostatic charge, and extracellular polymeric substances (EPS) production [41].

At the same time, for bacteria with more hydrophobic cell walls, attractive interactions between nonpolar fragments may increase their affinity toward fluorinated polyurethane [35]. Finally, the effectiveness of related strategies has also been confirmed in vivo studies. An ultrathin organosilane coating (MEG-OH) applied to a polyurethane catheter significantly reduced the number of bacteria, including *E. coli* and *S. aureus*, on the surface of the subcutaneously implanted device over a 30-day period [42]. However, in our case, such a pronounced increase in the water contact angle as reported in the above studies was not achieved. Consequently, plasma modification of polyurethane did not result in a uniform anti-adhesive effect across all tested strains. Although certain species, such as *E. coli*, exhibited greater surface coverage after modification, particularly after prolonged incubation, the effect was strain-dependent and not universal. In the present study, the increase in *E. coli* adhesion after plasma treatment is most likely related to the moderate increase in surface hydrophobicity (below the range typically associated with anti-adhesive fluorinated surfaces) together with the absence of pronounced micro/nanotexturing effects. Additionally, only early-stage adhesion (up to 240 min) was evaluated, which may differ from trends observed in long-term biofilm formation studies on highly fluorinated surfaces. This interpretation further supports the conclusion that the biological response observed in the present study depends not only on fluorination itself but also on the degree of hydrophobization and surface topography achieved. Overall, the applied plasma treatment altered bacterial adhesion in a strain-dependent manner rather than producing a consistent increase or decrease in colonization. At the same time, the overall AFM topography remained free of pronounced micron-scale features, suggesting that the modification mainly introduced irregularly distributed nanoscale surface asperities. This distinction is relevant for the interpretation of bacterial adhesion, because the observed roughness scale remains clearly below typical bacterial dimensions, i.e., approximately 1.0–2.0 µm for *E. coli* and 0.5–1.0 µm for staphylococci [43]. Therefore, the modified surface is unlikely to provide micron-scale shelters or geometric barriers directly matching the size of bacterial cells [44]. Instead, the literature indicates that nanoscale topography may influence early bacterial attachment indirectly, by modulating the accessible contact area, interfacial forces, and conditioning-film formation, and that the relationship between roughness and adhesion is often non-linear and species-dependent [43,45]. For example, model studies have shown increased adhesion at intermediate roughness values (RMS 10–40 nm) but markedly reduced adhesion at higher roughness (>45 nm), whereas other nanostructured systems demonstrated reduced bacterial attachment only for precisely defined nanoscale pores or architectures [46]. Therefore, the relationship between RMS roughness and bacterial adhesion is often non-linear [43]. Thus, in the present study, the observed strain-dependent adhesion pattern is more likely to reflect the combined effect of fluorine-induced surface chemistry and moderate nanoscale roughness than roughness alone.

It should be emphasized that the present study evaluated early-stage bacterial adhesion (up to 240 min), which constitutes the first and critical step in biofilm development. Stable attachment of planktonic bacteria to a surface determines subsequent microcolony formation and extracellular polymeric substance (EPS) production. Therefore, the observed strain-dependent differences in adhesion may influence later biofilm formation, although mature biofilms were not directly investigated in this study. Future work should include long-term incubation experiments to assess biofilm architecture and biomass on fluorinated polyurethane surfaces.

### 3.5. Biocompatibility

**Figure 5:**
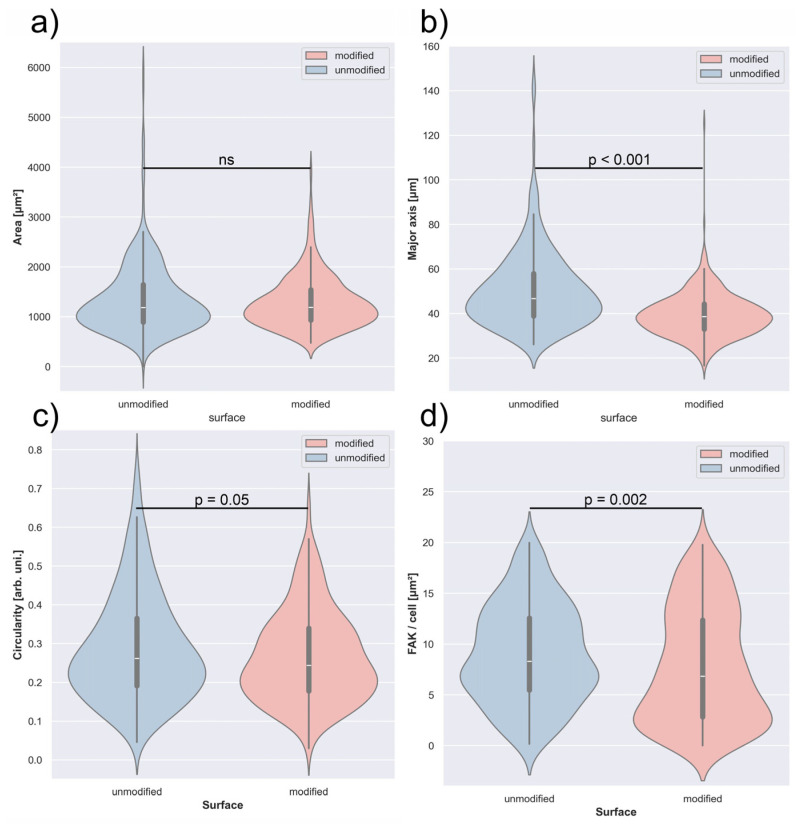
Distribution of cell morphological features—cell area (**a**), major axis length (**b**), circularity (**c**), and focal adhesion area per cell (**d**)—for A549 cells grown on unmodified and C_3_H_2_F_4_ plasma–treated polyurethane. Results are presented as violin plots displaying medians and interquartile ranges, where the median is represented by the white line.

To evaluate the effect of C_3_H_2_F_4_ plasma modification on A549 cell behavior, a detailed morphological and viability analysis was performed. Parameters related to cell spreading, shape, and adhesion were quantified and compared between modified and unmodified polyurethane surfaces. The analysis of A549 cell surface area revealed comparable results for both polyurethane types (Figure 5a). The median cell area was 1188.6 µm^2^ (Q1–Q3: 931.3–1543.2 µm^2^) on the modified surface and 1185.1 µm^2^ (Q1–Q3: 892.5–1646.7 µm^2^) on the unmodified one. No statistically significant difference was observed between the two groups (*p* = 0.958), indicating that C_3_H_2_F_4_ plasma modification did not notably influence the spreading of A549 cells in terms of occupied surface area.

The length of the major cell axis differed significantly between the two surface types (*p* < 0.001) (Figure 5b). Cells cultured on the modified polyurethane exhibited a median major axis length of 38.6 µm (Q1–Q3: 33.0–44.3 µm), whereas those on the unmodified surface reached 46.7 µm (Q1–Q3: 38.8–58.0 µm). These results indicate that cells on the unmodified polyurethane were more elongated.

Cell circularity showed only a slight difference between the two surface types (*p* = 0.05) (Figure 5c). The median circularity was 0.24 (Q1–Q3: 0.18–0.34) for cells on the modified polyurethane and 0.26 (Q1–Q3: 0.19–0.37) for those on the unmodified surface. Cells cultured on the modified polyurethane exhibited slightly lower circularity values compared to those on the unmodified surface

The analysis of FAK expression revealed a statistically significant difference between the two surfaces (*p* = 0.002) (Figure 5d). Cells grown on the modified polyurethane showed a median FAK signal of 8.88 µm^2^ (Q1–Q3: 3.37–17.14 µm^2^), while those on the unmodified surface exhibited higher values, with a median of 10.94 µm^2^ (Q1–Q3: 6.49–18.79 µm^2^).

**Figure 6:**
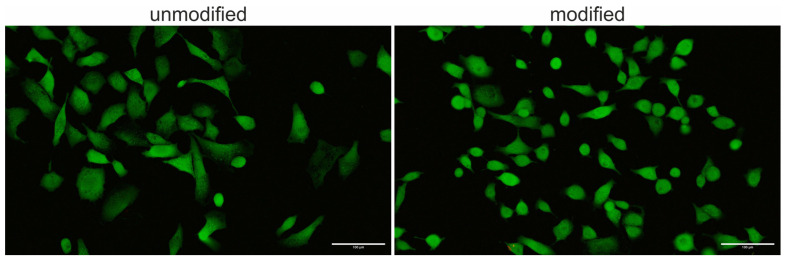
Biocompatibility evaluation of A549 cells cultured on unmodified and C_3_H_2_F_4_ plasma–modified polyurethane using FDA/PI staining. The white rectangle represents a scale bar of 100 μm.

Cell viability assessment of the A549 line using FDA and PI staining confirmed that neither the modified nor the unmodified biomaterial exerted a cytotoxic effect. Cells maintained a very high level of viability on both tested surfaces (Figure 6).

**Figure 7:**
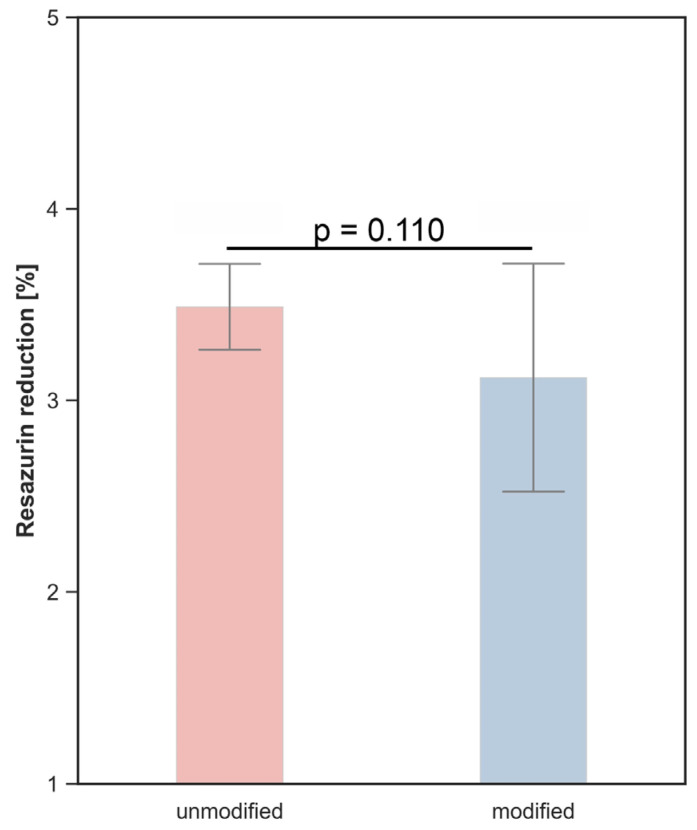
Metabolic activity (cell viability) of A549 cells cultured on unmodified and C_3_H_2_F_4_ plasma–modified polyurethane surfaces determined using the AlamarBlue reduction assay.

The AlamarBlue assay showed comparable metabolic activity of A549 cells cultured on unmodified and C_3_H_2_F_4_ plasma–modified polyurethane surfaces. The mean fluorescence intensity values were 3.49 ± 0.22 for the unmodified surface and 3.12 ± 0.59 for the modified surface. No statistically significant difference between the groups was observed (*p* = 0.110), indicating that plasma modification did not significantly affect A549 cell metabolic activity (Figure 7).

The ability of cells to adhere to and proliferate on biomaterial surfaces is strongly dependent on surface wettability. The most frequently cited “window” of optimal cell adhesion and proliferation corresponds to a water contact angle (WCA) of approximately 40–70°, reflecting moderate hydrophilicity [47]. At much higher contact angles, above 100°, approaching the level of superhydrophobicity, A549 cells exhibit minimal ability to colonize such surfaces. Studies conducted on silicon substrates with controlled wettability demonstrated that increasing the contact angle (and the presence of an air plastron between micro- and nanopillars) correlates with a gradual decline in cell density over time [48]. This phenomenon is consistent with the effect observed after C_3_H_2_F_4_ plasma treatment, which typically increases the contact angle (hydrophobization). However, in the present study, the water contact angle increased only moderately (from 92.6° to 97.9°) and did not reach the superhydrophobic regime. Therefore, although surface hydrophobization was observed, its magnitude was likely insufficient to induce a pronounced reduction in A549 cell attachment. Consequently, mechanotransduction of signals from focal adhesions (FAK) becomes weakened, and the cells adopt a more compact morphology, indicating reduced adhesive activity. If the goal is to achieve good adhesion of A549 cells, it is desirable to decrease hydrophobicity to a contact angle range of 40–70°. Conversely, when the aim is to minimize cell attachment (e.g., to prevent tissue colonization), maintaining high hydrophobicity (>100–120°) is advantageous; moreover, introducing micro/nanotextures further enhances the anti-adhesive effect [48]. Apart from wettability, surface topography is another crucial parameter determining cellular response. Micro- and nanoscale roughness significantly affect endothelial cell behavior by enhancing adhesion, spreading, and elongation compared to smooth surfaces. The highest adhesion is typically observed on microstructured or combined micro/nanostructured surfaces [49]. In the present study, AFM analysis demonstrated that C_3_H_2_F_4_ plasma modification preserved the global surface morphology while increasing nanoscale roughness, which may explain the subtle alterations in cell morphology without compromising overall cell viability.

## 4. Conclusions

C_3_H_2_F_4_ plasma modification of polyurethane enabled the introduction of -CF/CF_2_/CF_3_ functional groups, resulting in a slight increase in surface hydrophobicity and moderate alterations in nanoscale topography, without significant interference with the overall surface morphology. In bacterial adhesion assays, no clear trend was observed—the effect of modification depended on the bacterial strain and incubation time; however, enhanced adhesion of *E. coli* was noted. Biocompatibility analysis using A549 cells demonstrated good tolerance of the plasma-treated surface, with no signs of cytotoxicity and comparable cell spreading to the unmodified samples. These findings indicate that fluorination of polyurethane surfaces is a mild modification method that does not adversely affect the biological compatibility of the material and can be used to subtly tailor its surface properties. The method may be particularly useful where mild surface fluorination is required without compromising cell compatibility.

## Figures and Tables

**Table 1:** Deconvolution results of the C 1s, O 1s and F 1s XPS spectra for unmodified and C_3_H_2_F_4_ plasma-treated (modified) polyurethane surfaces.

Sample	Region	BE (eV)	Assignment	at.%
Unmodified	C 1s	285.08	C–C/C–H	81.0
		286.29	C–O–C	13.4
		287.7	C=O	5.6
	O 1s	531.44	C=O	31.8
		532.42	C–O/C–OH	29.1
		533.04	C–O–C	21.9
		533.68	C-O	12.0
		534.28	minor high-binding-energy O component	5.2
Modified	C 1s	285.83	C–C/C–H	42.8
		285.31	C–O–C	21.1
		287.88	C=O	15.7
		290.5	CF_2_	7.1
		292.94	CF_3_	13.4
	O 1s	532.03	C=O	20.1
		533.09	C–O/C–OH	41.4
		534.01	C–O–C	24.1
		534.75	O in CFx-influenced environment	10.1
		535.53	minor high-binding-energy O component	4.2
	F 1s	688.28	CFx (C–F bonds)	100

BE—binding energy; at.%—atomic percentage.

**Table 2:** Atomic concentration (at.%) of unmodified and plasma-treated (modified) polyurethane surfaces.

Surface	Atomic Concentration (at.%)			
	C 1s	O 1s	N 1s	F 1s
Unmodified	85.5	10.7	3.8	0
Modified	54.4	3.0	0.6	42.0

at.%—atomic percentage.

## Data Availability

The original contributions presented in this study are included in the article. Further inquiries can be directed to the corresponding author.
